# Military community engagement to prevent firearm-related violence: adaptation of project safe guard for service members

**DOI:** 10.1186/s40621-024-00490-9

**Published:** 2024-02-15

**Authors:** S. Rachel Kennedy, Jessica Buck-Atkinson, Jayna Moceri-Brooks, Megan L. Johnson, Michael D. Anestis, Makala Carrington, Justin C. Baker, Mary E. Fisher, Donald E. Nease, AnnaBelle O. Bryan, Craig J. Bryan, Marian E. Betz

**Affiliations:** 1 Department of Emergency Medicine, University of Colorado School of Medicine, 12401 E 17th Ave B-215, Aurora, CO 80045 USA; 2 Firearm Injury Prevention Initiative, University of Colorado Anschutz School of Medicine, Aurora, CO USA; 3 Injury and Violence Prevention Center, University of Colorado Anschutz Medical Campus, Aurora, CO 80045 USA; 4 New Jersey Gun Violence Research Center, Rutgers School of Public Health, Rutgers University, 683 Hoes Lane West, Piscataway, NJ 08854 USA; 5 Department of Psychiatry and Behavioral Health, The Ohio State University Wexner Medical Center, 1670 Upham Drive, Suite 130, Columbus, OH 43210 USA; 6 Department of Family Medicine, School of Medicine, University of Colorado Anschutz Medical Campus, 12401 E 17th Avenue B215, Aurora, CO 80045 USA; 7 Colorado Clinical and Translational Sciences Institute, Community Engagement and Health Equity, University of Colorado Anschutz Medical Campus, 1890 N Revere Ct, Campus Box B141, Aurora, CO 80045 USA

**Keywords:** Suicide, Firearm, Military, Lethal means, Community engagement

## Abstract

**Background:**

Suicide, especially by firearm, remains a leading cause of death in military populations in the USA. Reducing access to firearms, especially during high risk times, may help prevent suicide and other forms of violence. The purpose of this study was to adapt a promising existing lethal means safety intervention (Project Safe Guard, PSG) for cross-cutting violence prevention and peer support in active-duty service communities using community engagement methods.

**Methods:**

A two-pronged community-engaged research approach was employed, including the Community Translation (CT) process that engaged 15 Service Members from one installation to help adapt PSG successfully. In addition, qualitative data was collected from 40 active-duty service members and military violence prevention specialists through in-depth interviews and focus group discussions.

**Results:**

Qualitative data and CT feedback led to site-specific PSG adaptations. Participants emphasized the importance of peer-to-peer discussions and highlighted resource allocation, leadership support, and stigma on firearm ownership as potential implementation challenges.

**Conclusions:**

Findings demonstrate the feasibility of community-engaged research to adapt lethal means safety interventions within military populations. PSG implementation should consider resource allocation, leadership support, and addressing stigma. This study has implications for future policies and standards for performing research on sensitive topics, particularly among military populations.

**Supplementary Information:**

The online version contains supplementary material available at 10.1186/s40621-024-00490-9.

## Background

Suicide by firearm within the military continues to be a pressing public health concern and has been identified as a high priority by the Department of Defense (DoD). In 2020, firearms accounted for 67% of active-duty service member (ADSM) suicide deaths, 74% of Reserve suicide deaths, and 76% of National Guard suicide deaths (DOD 2022). Firearm access is associated with increased risk of suicide death (RAND 2018), and reducing firearm access during times of suicide risk (“lethal means safety”) is a focus of DoD and civilian suicide prevention strategies (DOD 2020; White House 2021).

Unfortunately, prior work suggests ADSMs with high suicide risk may be less likely to store home firearms securely (unloaded and locked), compared to those without high suicide risk (Anestis et al. 2021; Bryan et al. 2019). Further, firearm-owning ADSMs who did not disclose their suicidal thoughts/behaviors to others or who had not recently attended a behavioral health session were more likely to report storing home firearms unlocked (Anestis et al. 2023).

To date, lethal means safety and other interventions to promote secure firearm storage have often been focused on individuals with identified suicidal thoughts or behaviors, especially in clinical settings. A 2016 systematic review found 5 out of 7 firearm interventions to promote secure firearm storage between 2000 and 2012 targeted high-risk individuals exclusively within clinical settings (Rowhani-Rahbar et al. 2016). Yet many individuals may not disclose suicidality or be identified as at risk, suggesting firearm suicide prevention interventions should also be universal (i.e., directed toward broader populations) and outside of clinical settings (White House 2021).

Previously, Project Safe Guard (PSG) was developed and tested in a randomized controlled trial in the Mississippi National Guard (Anestis et al. 2021). PSG is a one-on-one brief intervention using motivational interviewing techniques to promote secure firearm storage practices among firearm owners in the military. During the trial, the intervention was delivered by public health graduate students to study volunteers (firearm owners in the National Guard). The study found that PSG was highly effective and acceptable: compared to the control group, participants who received the intervention adopted or improved secure firearm storage practices and maintained the changes at six months. Nearly all (99.6%) participants of the study also reported that they would recommend the intervention to other Service Members (Anestis et al. 2021).

Community Translation (CT) is a process designed to engage community members (referred to as the “CT Team”) to translate complex medical or research jargon, evidence-based guidelines, and recommendations into locally relevant actionable messages, interventions, and materials. In CT, community members meet regularly over a pre-defined time period for structured delivery of educational content on a topic and facilitated discussion about adaptation for specific community needs. It is a rigorous evidence-based process that has been replicated within many communities (Brewer et al. 2024; Curcija et al. 2022; Nease et al. 2018; Nease et al. 2018; Westfall et al. 2013). Building on the promising results of the PSG research study, we sought to use CT to adapt PSG for universal, peer-to-peer delivery in a “real-world” setting with ADSMs.

## Methods

We used a two-pronged community-engaged research approach including the (1) Community Translation (CT, also referred to as “Boot Camp Translation” in prior work) (Nease et al. 2018) process and (2) qualitative data collection. Specifically, the study team engaged installation community members to understand how to address limitations of the original PSG intervention. Key areas of exploration included how PSG could be (a) tailored to all ADSMs; (b) expanded in focus to various forms of firearm-injury and to peers; and (c) utilize a real-world delivery environment. Additionally, given the unique context of military life and culture, engagement of the base community was seen as paramount in developing an intervention and implementation plan that was culturally grounded and relevant to the local context.

This work built upon an existing relationship with a local military installation (US Space Force), and the project received official support from base leadership. While the base was engaged in various suicide and violence prevention activities, at the time of the study it had no formal firearm suicide prevention program or training. The study was approved by the Colorado Multiple Institutional Review Board and the Human Research Protection Office (HRPO).

### Community translation

CT Team members were recruited with the help of unit leadership and the command’s Violence Prevention Integrator using formal and informal communication channels. Recruitment strategies focused on recruiting ADSMs (military level E2-O4) and civilians involved in the delivery of other prevention programs or services. Meetings were in person and virtual; early meetings provided content background on the topic, with later meetings focused on the intervention and its adaptations. Meetings were facilitated by a study team member with CT experience. Study investigators were invited to the first CT meeting but intentionally not invited to subsequent meetings so they would not overly influence intervention adaptation. Meetings allowed for collaboration on the development of the adapted version of PSG, including the design of key visuals or written messages and the development of a roll-out plan for the intervention and assessment schedule. Participants who attended at least 75% of the meetings received incentives (swag items and letters of appreciation endorsed by base leadership). Additional depth discussion of CT methods is presented in a separate publication (Fisher et al., 2023, in review).

The final CT Team was made up of 15 representatives from the primary installation, including the Violence Prevention Integrator, and two members of the study team. The representatives from the installation were predominantly enlisted ADSMs (ranging in rank E3-E7) and male. The process occurred over the course of six virtual and 10 in-person meetings (February to November 2022).

### Qualitative methods

We oversampled ADSMs and branch leaders at the installation where the adaptation was taking place to participate in in-depth interviews (IDIs) and focus group discussions (FGDs). In addition, ADSMs and Violence Prevention Integrators from Army, Navy, and Air Force branches at other installations were recruited to gain insight into potential differences and similarities across branches. The study team worked closely with the Violence Prevention Integrators at both the primary installation and the other locations/branches to recruit and gain buy-in, with priority for recruitment to engage ADSM of all ranks for feedback and insight on PSG adaptation. All participants were English speaking adults. Per DoD policy, no incentives were offered to military participants on duty during their study participation.

Semi-structured FGDs and IDIs followed semi-structured guides (Additional file 1: Table S1) to gather information on: (1) ADSM experiences with prevention programs/messages, (2) PSG adaptation considerations, and (3) beliefs and behaviors surrounding secure storage of personal firearms. Informed consent was obtained prior to each interview. Interviews were conducted in person or by Zoom, and all interviews were recorded and transcribed verbatim through the independent transcription service Datagain (www.datagainservices.com). Transcripts were uploaded into qualitative data analytic software, Dedoose (www.dedoose.com) to facilitate team-based analysis, and all thematic coding was checked for accuracy and any discrepancies between the two coders. All qualitative results were triangulated with the CT results to inform adaptation discussions and decisions. For analysis, we used a mixed inductive (emergent) and deductive (hypothesis-driven) approach, as we have in prior work (Betz et al. 2013; Thomas et al. 2022).

A total of 40 participants were recruited during the qualitative study (March to October 2022). Participants included 15 ADSMs, 12 officers, 11 Violence Prevention Integrators, and 2 installation leaders. These 40 participants came from the Air Force (*n* = 6), Space Force (*n* = 28), Navy (*n* = 1), Army (*n* = 3), and Marines (*n* = 2).

## Results

### Community translation findings

Upon conclusion of the CT process, the team had developed (1) an adapted version of the PSG intervention, (2) recommendations for intervention roll-out within the installation itself, and (3) tailored messages and messaging products.

Overall, the CT Team had positive views of the PSG intervention and felt the approach offered, per one CT participant, a “process which leads participant(s) to safety but allows for them to define what that looks like by facilitating the creation of their own plan.” Additionally, the CT Team supported the intervention’s non-political approach and lack of connection to gun control policies, and it felt the focus on firearm safety and protecting one another during times of risk would resonate with the local community, regardless of individual firearm ownership. The CT Team felt it was important to highlight suicide prevention but also collectively endorsed expanding focus to address the prevention of other firearm harms (e.g., accidental shootings/child access). Further, the team agreed that the intervention would be a good addition to the existing prevention programs and efforts in place at the installation.

Final decisions from the CT Team on how to adapt PSG can be seen in Table 1. One of the most substantial modifications endorsed by the CT Team was the shift to universal delivery, meaning that roll-out, and therefore intervention design, needed to reach “total force”, including embedded civilians within each unit. This required the adapted version to be applicable to everyone within the installation—regardless of firearm ownership or access, level of risk, nor pre-existing mental health concerns. Other notable adjustments identified by the CT Team included the addition of an initial educational campaign (using team-developed materials; discussed below), use of peer facilitators, shortening of the facilitator training, and the provision of installation-specific information and resources during delivery. Many aspects of the PSG intervention were retained, notably, the one-on-one delivery method and the integration of motivational interviewing principles.

**Table 1:** PSG Adaptation feedback across methodologies

	Original PSG	CT adaptation suggestions	Qual feedback
Participants	Firearm-owning Mississippi National Guard members	“Total force”/universal delivery among all ADSM and embedded civilians, regardless of firearm ownership	“Total force”/universal delivery among all ADSM and embedded civilians, regardless of firearm ownership, as well as their families
Domain	Firearm suicide prevention (universal/upstream)	Secure firearm storage promotion to prevent variety of firearm-involved injuries and deaths	Secure firearm storage promotion to prevent firearm-involved injuries and deaths, including suicide, domestic violence, and child access
Topic	Self-protective environments—either all the time, or planning in case crisis arises	Self- and peer-protective environments, both all the time and planning in case crisis arises	Self- and peer-protective environments, both all the time and planning in case crisis arises
Facilitator	Study psychologists & psychologist trainees	Trained peers; unit members that are passionate about topic and respected by unit members	Trained peers and leaders; unit members that are passionate about topic and respected by unit members
Delivery setting	Lab-based delivery; private space	“Real world” during normal working hours; private, safe space	“Real world” during normal working hours; private, safe space
Method of delivery	Face-to-face, one-on-one	Face-to-face, one-on-one	Face-to-face, one-on-one
Dose/duration	Single session, 10–15 min	Single session, 10–15 min	Brief single sessions held regularly and made available as needed
Intervention components	Based on motivational interviewing	Maintain integration of motivational interviewing and encouragement of documenting planned behavior	Maintain integration of motivational interviewing and encouragement of documenting planned behavior
	Participant wrote down their behavior plan on a piece of paper at conclusion	Add educational “primer” campaign proceeding facilitator training and delivery of 1:1 conversation	Include hands-on activities such as taking participants to shooting ranges or creating family-friendly events
Facilitator training	Annual, 2 day workshop	4-h training	Ongoing fidelity monitoring
	Ongoing fidelity monitoring	Role-play/mock sessions included in training	
Participant materials	Cable locks provided (if assigned to that trial arm)	Lockboxes available to all participants	Secure locking device of participant’s choosing to all participants
		Installation-specific information and resources	

In addition to expanding the target audience beyond firearm owners, the CT team felt the primary message should focus on the role secure firearm storage can play in reducing firearm injury and death. Key messages the team wanted to convey included: (1) number of military suicides that involve firearms; (2) high likelihood of death by suicide with a firearm; (3) often short timeframe between contemplation and decision to attempt suicide; and (4) opportunity secure firearm storage provides to put time, space, and distance between someone experiencing heightened risk of harm and a highly lethal mean.

**Fig. 1:**
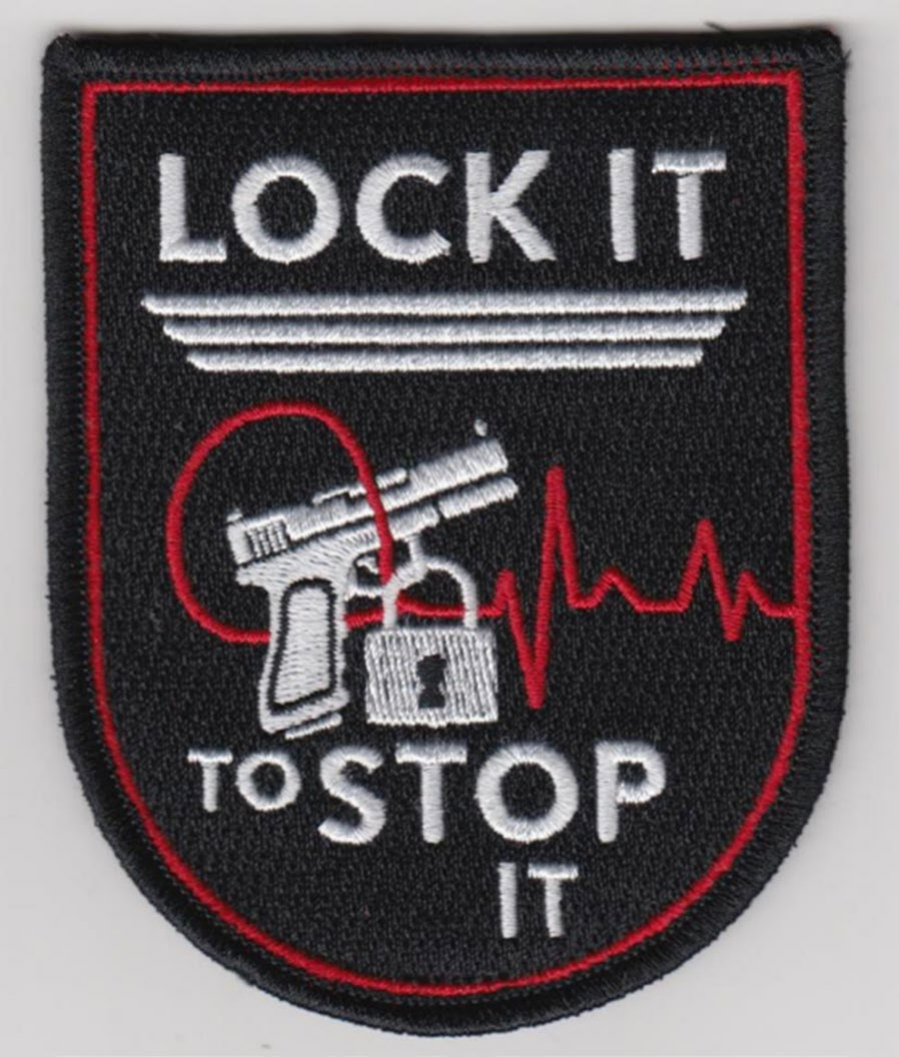
Lock it to stop it logo adapted by the community translation team

**Fig. 2:**
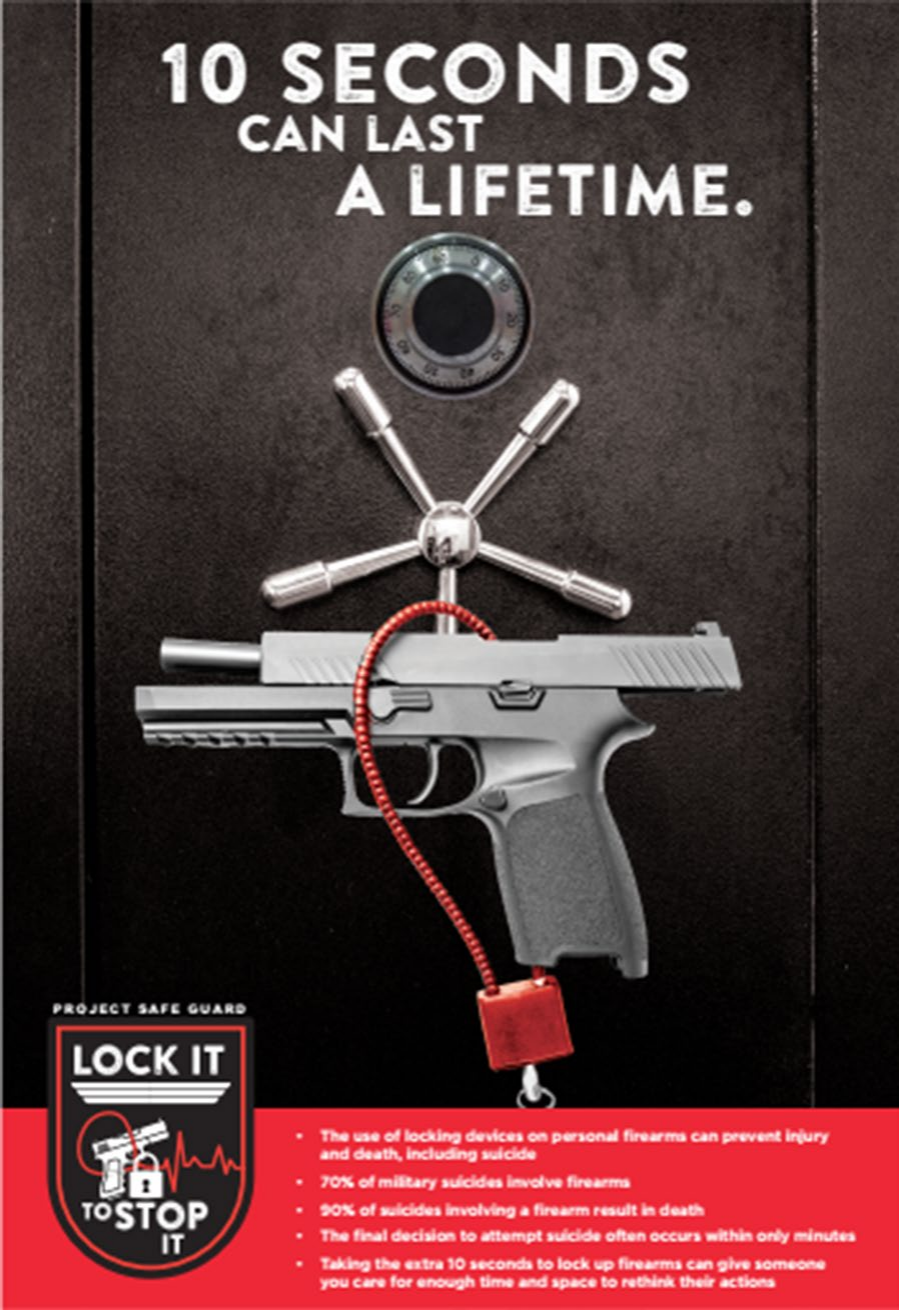
Educational flyer developed by the community translation team

These key concepts resulted in the creation of the slogan and corresponding logo (Fig. 1), “Lock It to Stop It”, which the CT Team chose to incorporate on an educational flyer alongside other key messages (Fig. 2). This slogan also led to the creation of a uniquely designed morale patch to be disseminated to the installation. When discussing the dissemination of these materials, the CT Team decided the materials would serve as an effective primer to the PSG intervention. The CT Team decided that regular, existing communication channels should be utilized for the flyers (e.g., placement in common locations like mess halls, restrooms, etc.) and that widescale distribution of the morale patches could help generate conversation and community attention.

### Qualitative results

Data from the qualitative IDIs and FGDs revealed the following themes: (1) areas of concern for firearm injury or death; (2) perceptions of how lethal means safety would be received and put into practice; (3) observations and feedback on existing firearm injury prevention efforts within the DoD; and (4) recommendations, feedback, and considerations for adapting PSG for the specific context of the partner installation.

Across all interviews, top areas of concern for ADSMs were firearm-involved suicide, domestic violence, and accessibility to children/others. While participants perceived suicide prevention as the highest priority to the DoD (versus domestic violence or child firearm access), they thought that ADSMs or their families may have different priorities.

Concerning lethal means safety (or firearm safety) messaging, participants discussed firearm storage and safety as being influenced by factors across individual, community, operational, and cultural levels of military life. The installation to which PSG was being adapted was seen as an empowering, collaborative community with many opportunities to incorporate a peer-led lethal means safety counseling intervention.

Stigma, funding, and staffing for prevention initiatives and mental health care were viewed as challenges to effective implementation of top-down prevention efforts. Thus, the peer-to-peer aspect of the proposed PSG intervention to promote secure firearm storage was well-received and thought to be an acceptable and feasible method for promoting lethal means safety among Service Members at the installation. As one participant noted, “I think it’s great… I personally just think that a lower level, more intimate conversation with folks would maybe just hit home more,” (Participant 29).

There were no major dislikes or concerns around introducing PSG to the community, and those from other branches noted similarities of PSG with violence/prevention efforts and best practices within their respective branches. Emphasis was placed on ensuring sufficient training, quality leadership, strong buy-in, and sufficient staffing/funds.

### Adapted PSG overview

The CT Team’s recommendations and qualitative findings were triangulated to shape the final adapted intervention. Key adaptations included shifting from a suicide prevention-centered approach to a broader firearm injury prevention approach, thereby also addressing domestic violence and child firearm access concerns. In addition, there was shift from having psychologists-in-training facilitate the intervention to having a peer-to-peer approach that would allow for ADSMs of all ranks, including lower enlisted, to serve as PSG facilitators if they were passionate about the topic and received appropriate training. PSG was adapted to be applicable to all branches (rather than only the National Guard), with a pre-intervention educational campaign to strengthen buy-in. Lastly, provision of more robust locking devices for participants, such as providing lockboxes rather than cable locks, was a key adaptation recommendation.

## Discussion

The original PSG intervention study, performed in a research setting in the National Guard, offered a promising example of an effective and acceptable universal intervention to increase secure storage of personal firearms in a specific military population (Anestis et al. 2021). The current study sought to expand the potential of PSG by adapting it for “real world” universal, peer delivery within the ADSM community. Expanding programming toward a universal approach for peer delivery to all individuals (not just those identified as “at risk”) may help change beliefs or behaviors before acute risk of violence develops, encourage lethal means safety interventions with at-risk peers, and normalize secure storage of personal firearms at all times. This type of upstream approach has the potential to reduce firearm-related suicide, interpersonal violence, and unintentional shootings.

Our use of a two-pronged community-based approach to guide the adaptation of PSG was grounded in previous research supporting the effectiveness of community-engaged research in producing effective, feasible, and acceptable interventions for communities (O’Mara-Eves et al. 2015). This approach allowed us to engage a diverse group of community members from the installation, which strengthened our ability to generate an intervention uniquely tailored to the local culture. It also revealed anticipated challenges and barriers that may impact intervention roll-out/implementation, allowing the study team to consider and preemptively address any concerns. Further, the use of community-based processes such as CT is novel within military populations, which offered an opportunity to explore their utility and feasibility for future use within military communities.

Our study was strengthened by the study team’s partnership with leadership at the partnered installation. The support for our efforts and the allocation of resources (e.g., personnel, meeting space, etc.) enhanced our ability to recruit diverse participants both for the CT Team and the qualitative study. Additionally, these partnerships helped the research team better understand the installation’s culture and context firsthand and facilitated trust between the study team and participants, which was crucial to project success.

There are several limitations to this work. Components of the adaptation process were not concurrent, but rather team decisions and discussion were conducted as data became available and team/participant availability allowed. While not necessarily a weakness, this limited the integration of feedback and suggestions in real-time. In addition, recruiting from other branches proved difficult compared to recruitment at the partner installation, including lack of buy-in from leaders at other installations and concerns around the confidentiality, nature, and privacy of these interviews.

## Conclusions

The community-engaged research approach of this study allowed adaptation of a promising intervention for use in active-duty military communities. It also generated recommendations for future large-scale dissemination and replication of locally-designed universal interventions across the DoD, including suggestions for uptake and sustainability. The close working collaboration with Service Members at a partner installation also laid the groundwork for future implementation and evaluation, which can contribute to public health implications and military readiness.

### Supplementary Information

**Additional file 1: Table S1.** Example interview guide used for qualitative interviews.

## Data Availability

Deidentified data underlying this article will be shared on reasonable request to the corresponding author.

## References

[CR1] Anestis MD, Bryan CJ, Capron DW, Bryan AO (2021). Lethal means counseling, distribution of cable locks, and safe firearm storage practices among the mississippi national guard: a factorial randomized controlled trial, 2018–2020. Am J Public Health.

[CR2] Anestis MD, Bond AE, Capron DW, Bryan AO, Bryan CJ (2023). Differences in firearm storage practices among United States military servicemembers who have and have not disclosed suicidal thoughts or attended behavioral health sessions. Suicide Life Threat Behav.

[CR3] Betz ME, Jones J, Petroff E, Schwartz R (2013). “I wish We could normalize driving health:” a qualitative study of clinician discussions with older drivers. J Gen Intern Med.

[CR4] Brewer SE, Fisher M, Zittleman L, Warman MK, Fort M, Gilchrist E (2024). Rapid Community Translation in the Colorado CEAL (CO-CEAL) Program: transcreating messaging to promote COVID-19 vaccination. Am J Public Health.

[CR5] Bryan CJ, Bryan AO, Anestis MD, Khazem LR, Harris JA, May AM (2019). Firearm availability and storage practices among military personnel who have thought about suicide. JAMA Netw Open.

[CR6] Curcija K, Zittleman L, Fisher M, Nease DE, Dickinson LM, de la Cerda D, et al. Does a rural community-based intervention improve knowledge and attitudes of opioid use disorder and medication-assisted treatment? A report from the IT MATTTRs study. J Rural Health Off J Am Rural Health Assoc Natl Rural Health Care Assoc. 2022;38(1):120–8. 10.1111/jrh.12545 PMC9290687 33244841

[CR7] DOD. DOD Instruction 6400.09. DOD policy on integrated primary prevention of self-directed harm and prohibited abuse or harm [Internet]. 2020. https://www.esd.whs.mil/Portals/54/Documents/DD/issuances/dodi/640009p.pdf?ver=2020-09-11-104936-223.

[CR8] DOD. DOD annual report on suicide in the military: calendar year (CY) 2021 [Internet]. Department of Defense; 2022. https://www.dspo.mil/Portals/113/Documents/2022%20ASR/Annual%20Report%20on%20Suicide%20in%20the%20Military%20CY%202021%20with%20CY21%20DoDSER%20(1).pdf?ver=tat8FRrUhH2IlndFrCGbsA%3d%3d.

[CR9] Nease DE, Daly JM, Dickinson LM, Fernald DH, Hahn DL, Levy BT (2018). Impact of a boot camp translation intervention on self-management support in primary care: a report from the INSTTEPP trial and Meta-LARC consortium. J Patient-Centered Res Rev.

[CR10] O’Mara-Eves A, Brunton G, Oliver S, Kavanagh J, Jamal F, Thomas J (2015). The effectiveness of community engagement in public health interventions for disadvantaged groups: a meta-analysis. BMC Public Health.

[CR11] RAND. The Relationship Between Firearm Availability and Suicide [Internet]. 2018. Available from: https://www.rand.org/research/gun-policy/analysis/essays/firearm-availability-suicide.html

[CR12] Rowhani-Rahbar A, Simonetti JA, Rivara FP (2016). Effectiveness of interventions to promote safe firearm storage. Epidemiol Rev.

[CR13] Thomas AC, Siry-Bove BJ, Barnard LM, Rooney L, McCarthy M, Mustafa A, et al. A qualitative study on diverse perspectives and identities of firearm owners. Inj Prev J Int Soc Child Adolesc Inj Prev. 2022;injuryprev-2022-044522. 10.1136/injuryprev-2022-044522 PMC9492625 35470245

[CR14] Westfall JM, Zittleman L, Sutter C, Emsermann CB, Staton EW, Van Vorst R (2013). Testing to prevent colon cancer: results from a rural community intervention. Ann Fam Med.

[CR15] White House. Reducing military and Veteran suicide: advancing a comprehensive, cross-sector, evidence-informed public health strategy [Internet]. 2021. https://www.whitehouse.gov/wp-content/uploads/2021/11/Military-and-Veteran-Suicide-Prevention-Strategy.pdf.

