# Supplementary material for: Military community engagement to prevent firearm-related violence: adaptation of project safe guard for service members
Source: Inj Epidemiol. 2024 Feb 15;11:7. doi: 10.1186/s40621-024-00490-9 (PMC10867994; doi:10.1186/s40621-024-00490-9)
Supplement: Supplementary file 1 — Additional file 1: Table S1. Example interview guide used for qualitative interviews. [file 40621_2024_490_MOESM1_ESM.docx]

**Supplement Table 1. Example interview guide used for qualitative interviews**

| **Question** | **Probes** |
| --- | --- |
| 1. A public health priority within the civilian context is to reduce the amount of firearm-related interpersonal violence, domestic violence, and self-injury (e.g., suicide). Which of these, if any, do you think are the biggest concern for members of the [military branch] and their families? | - 1. What do you see as the biggest area of concern for [military installation]?   2. What norms/expectations are there around this type of firearm-related injury or violence among the Service Members that you work with at [military installation]?   3. When these things are talked about, how are they discussed?   4. What kinds of factors or experiences do you think influence how Service Members think about firearm-involved injuries and death?   5. How do these factors influence their thinking, attitudes, and/or behavior?   6. Is it the same/different for other forms of firearm-involved injury or death (that we haven’t discussed)? |
| 1. What unique strengths/opportunities exist within the [military branch] to prevent firearm-involved injuries and death? | 1. Are there any additional strengths/opportunities that are unique to [military installation]? 2. How are Service Members empowered in their role to prevent these injuries/incidents from the bottom to top? Top down? (*room for interpretation) 3. Are these the same/different for other forms of firearm-involved injury or death (that we haven’t discussed)? |
| 1. What unique challenges/barriers existing within the [military branch] to prevent firearm-involved injuries and death? | - 1. What makes it difficult to actively address firearm safety, interpersonal violence prevention, and/or self-harm/suicide prevention to Service Members at [military installation]?   2. Are there any additional challenges/barriers that are unique to [military installation]?   3. Are these the same/different for other forms of firearm-involved injury or death (that we haven’t discussed)? |
| 1. Do you think Service Members at [military installation] feel they play a role in preventing firearm-related injuries and violence? | - 1. How do you they feel they do/do not play a role?   2. In what ways have you seen Service Members play/carry out this role?   3. Is it the same/different for other forms of firearm-involved injury or death (that we haven’t discussed)?   4. In what additional ways do you think Service Members can play a role?   5. Are there are certain types of firearm violence that are easier to discuss among Service Members compared to others? |
| 1. What effective programs/messages around firearm-related violence prevention have you seen delivered in the past with Service Members? | - 1. Who has delivered these programs/messages?   2. Where have they been delivered/what method of delivery has been used?   3. Who is the typical target audience for these programs/messages? Do these programs/messages reach their intended audience?   4. What was successful about this program/message?   5. What was unsuccessful about this program/message?   6. What would have made this program/message more successful or effective? |
| 1. What ineffective programs/messages around firearm-related violence prevention have you seen delivered in the past with Service Members? | 1. Who has delivered these programs/messages? 2. Where have they been delivered/what method of delivery has been used? 3. Who is the typical target audience for these programs/messages? Do these programs/messages reach their intended audience? 4. What was successful about this program/message? 5. What was unsuccessful about this program/message? 6. What would have made this program/message more successful or effective? |
